# Wrist Fracture in a Child Irreducible Due to Soft Tissue Interposition

**DOI:** 10.5811/cpcem.2018.7.38952

**Published:** 2018-08-16

**Authors:** Shinsuke Takeda, Katsuyuki Iwatsuki, Akihiko Tabuchi, Sadahiro Kubo, Satoshi Teranishi, Hitoshi Hirata

**Affiliations:** * Anjo Kosei Hospital, Emergency and Critical Care Center, Anjo, Japan; † Nagoya University Graduate School of Medicine, Department of Hand Surgery, Nagoya, Japan

## CASE PRESENTATION

**Image 1:**
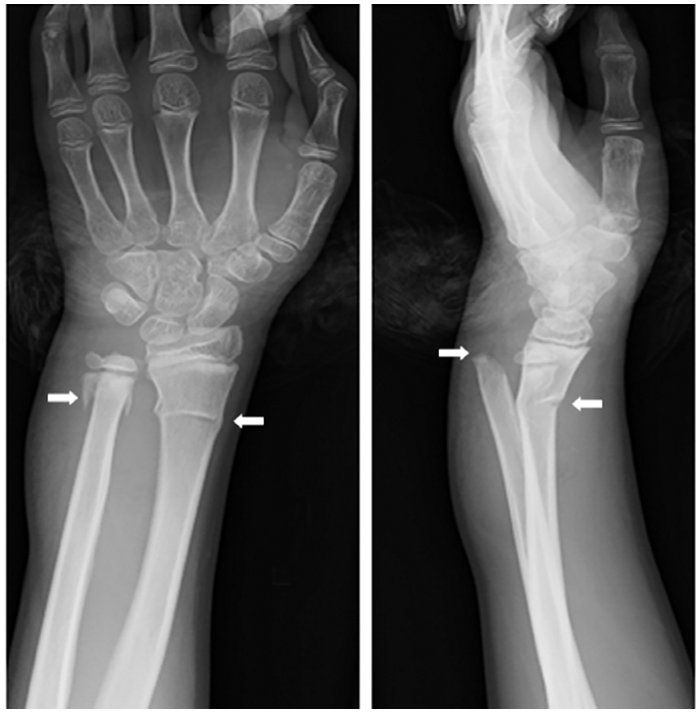
Anteroposterior (left) and lateral (right) radiographs of the distal forearm and wrist joint. Radiographs show the radius and ulnar fractures (arrows).

An 11-year-old boy fell onto his outstretched arm. He presented to the emergency department with a deformity of his left wrist. Radiograph revealed a greenstick fracture with volar angulation of the distal radius. The distal ulnar physis was disrupted (Salter-Harris type II) and the proximal metaphyseal fragment was displaced dorsally; however, the distal radioulnar joint was intact (Image 1). Closed reduction of the distal ulna under axillary block failed. Three-dimensional computed tomography (3DCT) was performed before open reduction.

## DIAGNOSIS

**Image 2:**
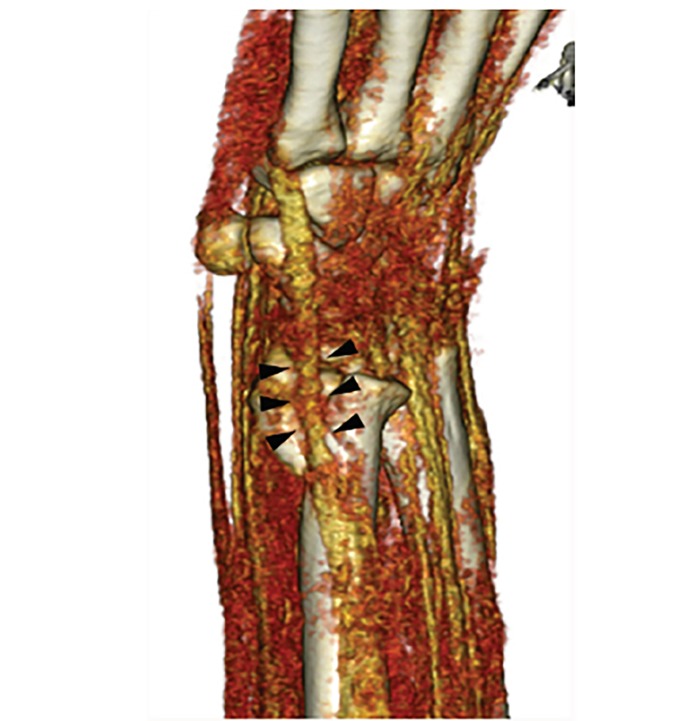
Three-dimensional computed tomography shows the interposition of the extensor carpi ulnaris between the fragments (arrows).

This type of fracture is known as a Galeazzi-equivalent fracture. Galeazzi-fracture dislocation is a well-known injury, consisting of a distal radial shaft fracture and dislocation of the distal radioulnar joint (DRUJ). It is rare in adults, and even more uncommon in children. In contrast, Galeazzi-equivalent fractures consist of a fracture at the distal radial metadiaphyseal area with complete distal ulnar epiphyseal separation instead of the more common pattern of DRUJ dislocation.^1^ The ulnar physeal fracture in a Galeazzi-equivalent injury can be irreducible due to soft tissue interposition (periosteum, extensor tendons, or joint capsule). It is important to identify and analyze these fractures precisely, as growth arrest has been reported after such injuries.^2^ 3DCT revealed the interposition of the extensor carpi ulnaris between the fragments, which hindered the reduction; this was confirmed intraoperatively (Image 2). The patient required open reduction and fixation of the ulnar physeal fracture with two Kirschner wires. He has regained wrist range of motion, with no complications at two-year follow-up.

**CPC-EM Capsule**

What do we already know about this clinical entity?

The ulnar physeal fracture in a Galeazzi-equivalent fracture can be irreducible due to soft tissue interposition, such as periosteum, extensor tendons, or joint capsule.

What is the major impact of the image(s)?

The interposition of the extensor carpi ulnaris (ECU) between the fragments, which hindered the reduction was revealed by three-dimensional computed tomography (3DCT).

How might this improve emergency medicine practice?

This case report reveals the ECU interposition of this Galeazzi-equivalent fracture by 3DCT and shows the difficulty of closed reduction in the emergency department.

Documented patient informed consent and/or Institutional Review Board approval has been obtained and filed for publication of this case report.
